# An inhomogeneity correction algorithm for irregular fields of high‐energy photon beams based on Clarkson integration and the 3D beam subtraction method

**DOI:** 10.1120/jacmp.v7i1.2042

**Published:** 2006-02-21

**Authors:** Sotirios Stathakis, Constantin Kappas, Kiki Theodorou, Nikos Papanikolaou, Jean‐Claude Rosenwald

**Affiliations:** ^1^ Radiation Oncology Department Fox Chase Cancer Center Philadelphia Pennsylvania 19111 U.S.A.; ^2^ Medical Physics Department Medical School University of Thessalia Larisa Hellas; ^3^ Department of Radiation Oncology University of Arkansas for Medical Sciences Little Rock Arkansas 72205 U.S.A.; ^4^ Institut Curie Section Medicale 26 Rue d’ Ulm 7500 Paris France

**Keywords:** inhomogeneity correction, radiation therapy, photons

## Abstract

A number of treatment‐planning systems still use conventional correction methods for body inhomogeneities. Most of these methods (power law method, tissue—air ratio (TAR), etc.) consider only on‐axis points, rectangular fields, and inhomogeneous slabs covering the whole irradiating field. A new method is proposed that overcomes the above limitations. The new method uses the principle of the Clarkson method on sector integration to take into account the position and lateral extent of the inhomogeneity with respect to the point of calculation, as well as the shape of the irradiating field. The field is divided into angular sectors, and each sector is then treated separately for the presence of inhomogeneities using a conventional correction method. Applying this method, we can predict the correction factors for Co‐60 and 6‐MV photon beams for irregular fields that include inhomogeneities of lower or higher densities relative to water. Validation of the predicted corrections factors was made against Monte Carlo calculations for the same geometries. The agreement between the predicted correction factors and the Monte Carlo calculations was within 1.5%. In addition, the new method was able to predict the behavior of the correction factor when the point of calculation was approaching or moving away from the interface between two materials.

PACS number(s): 87.53.Bn, 87.53.Wz

## I. INTRODUCTION

Traditionally, the clinical experience with radiation therapy has been based on the tissue responses to the planned doses in a homogeneous body even if the irradiated volume contains inhomogeneous volumes (mainly lungs, air cavities, and bones).

The dramatic increase in computing power at affordable prices has greatly enhanced several technical advances in radiotherapy. The radiotherapy treatment‐planning system (RTPS) that uses 3D patient data is a reality. Several algorithms have been proposed to implement some sort of inhomogeneity correction, from the simplified tissue—air ratio (RTAR), which yields a correction factor for water‐based calculations, to superposition/convolution and Monte Carlo methods, which include the inhomogeneity in the calculation of patient dose. At present, the majority of the current commercial RTPS offer the equivalent TAR (ETAR)^(1)^ method and other conventional methods developed more than 20 years ago^(^ ^2^ ^–^ ^4^ ^)^ as inhomogeneity correction algorithms. The shortcomings of these earlier methods are well known, and sometimes calculation differences of 10% from measurements are not uncommon.

The *conventional* methods, such as the power law method, RTAR, and differential TAR (DTAR),^(^ ^5^ ^–^ ^8^ ^)^ of inhomogeneity corrections in high‐energy X‐ray beams assume that

1. the points where the primary dose is altered by the presence of inhomogeneity are considered to be centered on the beam axis, and the lateral dimensions of the inhomogeneity being larger than the field dimensions;
2. for the points where the primary dose is not altered, the correction factor is taken as unity.

In addition, these methods are not able to directly take into account the shape of the irradiating field (irregular fields).

The most common inhomogeneity method is the Batho method for which several improvements have been proposed. Such improvements were focused on correcting the extent of the inhomogeneity as well as the position of the point of interest. Wong and Purdy^(9)^ have shown that modification of the Batho power law method into an additive form would improve its application. El‐Khatib and Battista^(8)^ replaced TAR values by tissue maximum ratio (TMR) values in the power law Batho method for cobalt‐60 and found marked improvement by nearly 5% in the accuracy of dose calculated within the lung. This was confirmed by numerical comparison of the Batho expression with an analytic solution of the primary and first‐scattered radiation. In addition, Yuen and Kornelsen^(10)^ have shown that the differential Batho method can give good results for circular fields where annulus slabs of inhomogeneity are inserted. Woo et al.^(11)^ proposed a new method of primary scatter separation that improves the dose calculations when used with the ETAR method. Kappas and Rosenwald^(12)^ proposed a method that takes into account the relative position of the point of calculation to the inhomogeneity as well as the lateral extent of the inhomogeneity. However, it should be mentioned that the literature concerning bulk correction methods for inhomogeneities lying inside irregular fields is limited.

The majority of the RTPS offer the option of the ETAR method as the primary inhomogeneity correction method. It has been shown, though, that the ETAR method yields results similar to the Batho method for single photon beams traversing geometries with inhomogeneities except with small inhomogeneities, where the ETAR method is more accurate than the Batho method. Moreover, there are several older RTPS still in use that offer the option of using only conventional methods. Hence, we propose a new approach based the principles of the Clarkson method of scatter integration^(13)^ and the 3D beam subtraction method (3D‐BSM),^(12)^ which could be used for irregular fields and any shape of inhomogeneous structures. The basis of this new method is to use the Clarkson principle so that each sector will be a circular field that has or does not have an inhomogeneity embedded inside, and the point of calculation is always at the central axis of the circular field in order to fulfill the requirements of the conventional methods. Unlike the original Clarkson method, in our approach we sum both primary and scatter dose in the sector integration. Then we apply the 3D‐BSM principle, which uses a mathematical combination of on‐axis conventional correction factors, each of which is calculated according to the actual size and position of the inhomogeneity relative to the calculation point.

Splitting the irradiating field into sectors according to the Clarkson principle (for each calculation point) and applying the concept of the 3D‐BSM method of computing the dose for each sector as a sum of “theoretical fields” leads to a “general correction factor” (combination of the corrections of all the individual sectors). Unlike most conventional methods, with this approach we consider (1) the projection of the shape of the inhomogeneity to the calculation plane and the size and position of the inhomogeneity inside the irradiating field, (2) the position of the calculation point, and (3) the shape of the irradiating field.

## II. MATERIALS AND METHODS

### A. Review of the 3D‐BSM

**Figure 1:**
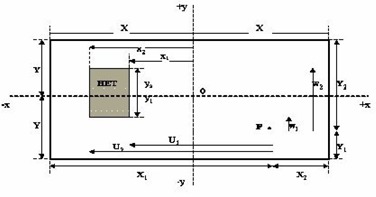
Case of a rectangular inhomogeneity in a rectangular field of dimensions 2*x* and 2*y*. $U_{i}$ and $W_{j}$: distances from point P to the inhomogeneity limits $x_{1},\;x_{2}$ and $y_{1},\;y_{2}$. $U_{i}=x_{i}-x_{p},\;W_{j}=y_{j}-y_{p}$

The 3D‐BSM was developed by Kappas and Rosenwald.^(12)^ Their method is based on the Day method^(1)^ for rectangular fields. Consider the rectangular field in the beam's‐eye view (BEV) (Fig. 1). Inside the irradiating volume is a parallelepiped of inhomogeneous volume and a calculation point, P. According to this method, the algebraic distances from the point of calculation to the limits of the inhomogeneity are calculated. Then the initial rectangular field can be split into sections (theoretical beams), where each section is made from the above calculated distances so that

1. each individual section contains the point P, and
2. each section is made only of one specified density (either water equivalent or inhomogeneity).

Then the dose to the point of calculation can be expressed as a sum of these theoretical beams using the Day method. This leads to a general correction factor (GCF):

(1) $$\text{GCF}=1+\frac{\sum_{i=1}^{2}{\left(-1\right)}^{i}\sum_{j=1}^{2}{\left(-1\right)}^{j}[\varepsilon_{ij}^{\prime }(C_{ij}-1)∙D_{o}^{w}(U_{i},W_{j})]}{\sum_{i=1}^{2}\sum_{j=1}^{2}\varepsilon_{i}D_{o}^{w}(X_{i},Y_{j})}$$

where $\varepsilon_{\text{ij}}$ is the sign of the dot product $\left(U_{i},\;W_{j}\right)$,^(12)^ $C_{\text{ij}}$ is the correction factor for each of the theoretical beams created, $D_{o}^{w}$ is the dose to water for the each field of dimensions defined in the parentheses, $U_{i}$ and $W_{j}$ are the algebraic distances from the point of calculation to the inhomogeneity limits, and $X_{i}$ and $Y_{j}$ are the distances from the point of calculation to the field boundaries.

The correction factor $C_{\text{ij}}$ could be calculated with a standard method such as the power law Batho method, which gives good results.^(^ ^8^ ^,^ ^9^ ^)^

### B. 3D‐BSM applied to irregular fields and irregular inhomogeneities

**Figure 2:**
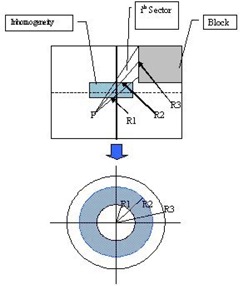
The BSM applied for each sector for a rectangular field with blocks shaping the field

The proposed method is based on the principle of the 3D‐BSM where the dose at a point is calculated as the algebraic summation of theoretical beams. The combination with the Clarkson method of sector integration allows the calculation of the dose at any point in the irradiating volume, taking into account the position and shape of the inhomogeneity relative to the point of calculation, as well as the shape of the field (Fig. 2). Consider Fig. 2, where the irradiating field, in BEV, is divided into angular sectors of φ degrees (the origin of the sectors is the calculation point P). Such sectors may contain a portion, all, or none of the inhomogeneity, and the dose to the point of calculation will be the sum of the contributions of all the sectors.

The goal is to correct the dose contribution from all sectors that include inhomogeneities. For every sector that intersects the inhomogeneities, the distances from the point of calculation to the proximal and distal boundaries of the inhomogeneities are calculated. This way, concentric fields are created that can be thought as if they are irradiating uniform media of density ρ. The density ρ of these media is associated with the corresponding radius of the field. For example, in Fig. 2 for the *i*th sector, the field of radius $R_{1}$ will irradiate the homogeneous medium of density $\rho_{1}$, the field of radius $R_{2}$ will irradiate the homogeneous medium of density $\rho_{2}$, and the field of radius $R_{3}$ will irradiate the homogeneous medium of density $\rho_{1}$. For such concentric fields, the dose can be corrected using any bulk correction method such as the power law (Batho), RTAR, etc. From now on, we will refer to these correction methods as “internal correction methods” and the corresponding correction factors as “internal correction factors,” since they are applied to each sector individually. The contribution of each such sector is proportional to the total number of sectors, given that all sectors have the same angle φ For points of calculation that do not lie along the beam central axis, we assume that the field is flat and no off‐axis ratios are considered in the calculations. For each sector one can algebraically add/subtract the circular fields created for any given sector in order to account for the perturbation of the dose for this sector. The same procedure should be followed for all the sectors until the completion of a 360° rotation. It should be clear that more than one inhomogeneity could be included in any sector. In such cases, the number of radii that have to be computed for these sectors will be increased accordingly.

Two cases are presented below to demonstrate the principles of the algorithm.

#### B.1 Case 1

Let us assume the rectangular field given in BEV (Fig. 2). The irradiated medium is water equivalent (density $1\;g/{\text{cm}}^{3}$) containing an inhomogeneity slab of density $\rho \;g/{\text{cm}}^{3}$ relative to water. For simplicity, assume that the inhomogeneity is included only in the *i*th sector. The dose from each sector to the point of calculation P can be computed using a Clarkson integration technique. The contribution of the dose from the sector *i* that intersects the inhomogeneity is given as

(2) $$D{\left(\rho ,R\right)}_{i}=\frac{\varphi }{360^{\circ }}(D(w,R_{3})-D(w,R_{2})+D(\rho ,R_{2})-D(\rho ,R_{1})+D(w,R_{1}))$$

where $D{\left(\rho ,R\right)}_{i}$ is the dose contribution from the *i*th sector to the point of calculation $P,\;D(\rho ,R_{1})$ are $D(\rho ,R_{2})$ are the doses of the circular fields of radii $R_{1}$ and $R_{2}$ containing inhomogeneity of density ρ relative to water, $D(w,R_{1}),\;D(w,R_{2})$, and $D(w,R_{3})$ are the doses from the circular fields of radii $R_{1},\;R_{2}$, and $R_{3}$ in water (*w*), and φ is the angle of the sector in degrees. The same can be applied for the rest of the sectors not intersecting an inhomogeneity; therefore, the total dose to the point $P\;D\;{\left(\rho ,R\right)}_{\text{Total}}$ can be calculated as

(3) $$D{\left(\rho ,R\right)}_{\text{Total}}=\frac{\varphi }{360^{\circ }}\left[\frac{360^{\circ }}{\varphi }D{\left(w,R\right)}_{\text{Total}}+D(w,R_{2})(C(\rho ,R_{2})-1)+D(w,R_{1})(1-C(\rho ,R_{1}))\right]$$

$D_{p}{\left(w,R\right)}_{\text{Total}}$ is the total dose from all sectors to the same point P in water. (In Eq. (3) we assume that one sector encloses the entire inhomogeneity.) $C(\rho ,R_{1})$ and $C(\rho ,R_{2})$ are the correction factors for the circular fields of radius $R_{1}$ and $R_{2}$, respectively, irradiating medium of ρ density.

If we try to generalize the above and get a GCF for the point P from all sectors,

(4) $$\text{GCF}=\frac{D{\left(\rho ,R\right)}_{\text{Total}}}{D{\left(w,R\right)}_{\text{Total}}}$$

We can write Eq. (4) using Eq. (3) and get the GCF for this example:

(5a) $$\text{GCF}=\frac{\frac{\varphi }{360^{\circ }}\left[\frac{360}{\varphi }D{\left(w,R\right)}_{\text{Total}}+D(w,R_{2})(C(\rho ,R_{2})-1)+D(w,R_{1})(1-C(\rho ,R_{1}))\right]}{D{\left(w,R\right)}_{\text{Total}}}$$

We can write Eq. (5a) as

(5b) $$\text{GCF}=1+\frac{\varphi }{360^{\circ }}\frac{D(w,R_{2})[C(\rho ,R_{2})-1]+D(w,R_{1})[C(\rho ,R_{1})]}{D_{p}{\left(w,R\right)}_{\text{Total}}}$$

Equation (5b) is valid when the inhomogeneity is anywhere in the field and is included in one sector of φ/360°.

#### B.2 Case 2

**Figure 3:**
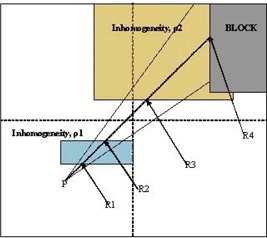
Case 2 beam's‐eye view of the BSM applied for a sector containing two inhomogeneities with the last one adjacent to the edge of the field

Now let us assume the irregular field in Fig. 3, where there are two inhomogeneity slabs in the field. For simplicity, we assume that both inhomogeneities are intersected by only one sector. The medium is water, and the two slabs have densities $\rho_{1}$ and $\rho_{2}$ relative to water, respectively. The dose from each sector to the point of calculation P can be calculated as in the previous example. The contribution of the dose from the sector *i* that intersects both the inhomogeneous slabs is given as

(6) $$D{\left(\rho ,R\right)}_{i}=\frac{\varphi }{360^{\circ }}\left(\begin{array}{l} D(\rho_{2},R_{4})-D(\rho_{2},R_{3})+D(w,R_{3})-D(w,R_{2})+\\ +D(\rho_{1},R_{2})-D(\rho_{1},R_{1})+D(w,R_{1}) \end{array}\right)$$

where $D{\left(\rho ,\;R\right)}_{i}$ is the dose contribution from the *i*th sector to the point of calculation $P,\;D(\rho ,\;R_{4}),\;D(\rho ,\;R_{3}),\;D(\rho ,\;R_{2})$, and $D(\rho ,\;R_{1})$ are the doses from the circular fields of radii $R_{1},\;R_{2},\;R_{3}$, and $R_{4}$ containing inhomogeneity of relative density $\rho_{1}$ or $\rho_{2}$, and $D(w,\;R_{1}),\;D(w,\;R_{2})$, and $D(w,\;R_{3})$ are the doses of the circular fields of radii $R_{1},\;R_{2}$, and $R_{3}$ of a water‐like medium, respectively. φ is the angle of the sector in degrees.

Following the same algorithm as in the previous example and taking into account the geometry of our example, we can calculate the total dose to the point P and therefore the GCF, assuming that only one sector intersects the geometry, as

(7) $$\text{GCF}=1+\frac{\varphi }{360^{\circ }}\left[\frac{\begin{array}{l} D(w,R_{4})(C(\rho_{2},R_{4})-1)+D(w,R_{3})(1-C{\left(\rho_{2},R_{3}\right)}_{3}^{\rho_{2}})+\\ D(w,R_{2})(C(\rho_{1},R_{2})-1)+D(w,R_{1})(1-C(\rho_{1},R_{1})) \end{array}}{D{\left(w,R\right)}_{\text{Total}}}\right]$$

#### B.3 Generalization of the correction factor

Let us consider Eqs. (5) and (7). If the inhomogeneities span over two, the second parts of Eqs. (5b) and (7) would be written as a sum of two terms so that each of them would take into account each sector. In a generalized form, the second part of Eqs. (5b) and (7) can be represented as a sum, where the number of the terms is equal to the number of the sectors that intersect the inhomogeneity. Equations (5b) and (7) can be written in a more general form for all cases:

(8) $$\text{GCF}=1+\frac{\varphi }{360^{\circ }}\frac{\sum_{i=1}^{n}\left[\sum_{j=1}^{m-1}D_{i}(w,R_{j})[C_{i}(\rho_{j},R_{j})-C_{i}(\rho_{j+1},R_{j})]+D_{i}(w,R_{\max})[C_{i}(\rho_{\max},R_{\max})-1]\right]}{D{\left(w,R\right)}_{\text{Total}}}$$

where *n* is the number of sectors intersecting the inhomogeneities, and *m* is the number of intersections between the point of calculation and the boundary of the irradiating field due to the presence of inhomogeneities. $D_{i}(w,\;R_{j})$ and $C_{i}(\rho_{j},\;R_{j})$ are the dose to the point of calculation and the correction factor of that dose, respectively. The subscript *i* denotes the sector, and the subscript *j* denotes the number of intersections from the point of calculation to the field boundaries. The first term in the parentheses accounts for the medium that the dose or the correction factor is applied for, and the second term denotes the radius of the field. The subscript “max” in Eq. (8) is used for the largest radius, which is the distance from the point of calculation to the field limits, and for the density $\left(\rho_{\max}\right)$ of the medium that is lastly traversed from that radius.

As one can see, if the inhomogeneity forms an “island” inside the irradiating volume (in BEV) of water and the point of calculation is not below or inside it, then the last term of Eq. (8) is zero because $C_{i}(\rho_{\max},\;R_{\max})=1$ (no correction is necessary when the medium is water). In the example of Fig. 2 we should have that $C_{i}(\rho_{\max},\;R_{\max})=1$, since the medium after the inhomogeneity is water. In this example we also have n=1,m=3. Then application of Eq. (8) yields

(9) $$\text{GCF}=1+\frac{\varphi }{360^{\circ }}\frac{D(w,R_{2})[C_{1}(\rho_{2},R_{2})-C_{1}(\rho_{3},R_{2})]+D(w,R_{1})[C_{1}(\rho_{1},R_{1})-C_{1}(\rho_{2},R_{1})]}{D{\left(w,R_{1}\right)}_{\text{Total}}}$$

The correction factors for the fields of radii $R_{1}$ and $R_{2}$ correcting for the first and third inhomogeneity are $C_{1}(\rho_{1},\;R_{1})$ and $C_{1}(\rho_{3},\;R_{2})$. In this case, $\rho_{1}$ and $\rho_{3}$ are water; therefore, the correction factors are equal to 1. Hence, Eq. (9) becomes Eq. (5b).

For the second case of Fig. 3, application of Eq. (8) for the point P inside a water‐like medium $\left(\rho =1\;g/{\text{cm}}^{3}\right)$, assuming that only one sector intersects the inhomogeneities, we have n=1,m=4, and $\rho_{1},\;\rho_{2}$ are the densities of the inhomogeneities relative to water, yielding again Eq. (7). From now on, we will call the new method 3D BSM IRREG.

### C. Development of test code

We implemented a test code for the method described above in order to validate Eq. (8) in various conditions. The implementation was made using C++. A simple user interface was developed where the user can enter the points that define the shape of the irregular field at source‐to‐axis (SAD)=100cm in Cartesian coordinates having the center of the field at the point (0, 0). The user also enters the points that determine the inhomogeneity shape in BEV, the depths of the upper and lower surface of the inhomogeneity, and the depth of the point of measurement. For any given depth of the point of interest, its x‐ and y‐coordinates are required. Points outside the field dimensions are not considered. The code is able to take into account the increase of the distance from the point of measurement to the source as the point of interest moves away from the origin (0, 0). The Batho method is the internal correction method in our implementation $\left(C_{i}(\rho_{j},\;R_{j}\right)$). Two sets of depth data were used: one for a cobalt‐60 machine taken from the British Journal of Radiology tables^(14)^ and one from measurements in water of our department's 6‐MV Philips SL75‐5 LINAC. Different depth dose data depending on the energy of the beam can be loaded by choosing the corresponding databases.

It should be mentioned that methods other than the Batho method of inhomogeneity correction could be applied as internal correction factors, since these routines are independent from the geometry routines. In the future, the code will have the option of using more correction methods, such as the DTAR, RTAR, etc., and more sets of dosimetric data for various energies.

## III. RESULTS

### A. Benchmark simulation

First, the new method was compared against the original BSM method with the same experimental setup (for rectangular fields) as described in the paper by Kappas and Rosenwald,^(12)^ and the results were in agreement with a minor discrepancy (0.5%), which can be attributed to round‐up errors in the interpolation routines.

**Figure 4:**
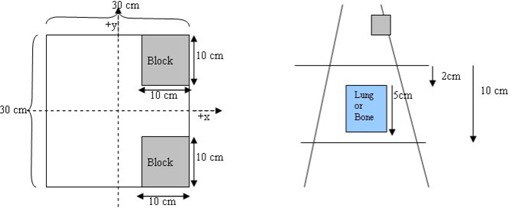
Setup geometry for the experiment. The dimensions of the blocks when projected to the level of the point of interest are 10cm×10cm.

Then, in order to verify the validity of Eq. (8), the setup geometry of Fig. 4 was simulated using the BEAM‐OMEGA^(15)^ code. An MDS Nordion Co‐60 machine and the 6‐MV Philips SL75‐5 LINAC were simulated in order to provide the necessary phase spaces for dose calculations.

First, the phase space files for the $10\times 10\;{\text{cm}}^{2}$ were created for both machines, and the dose distributions were calculated in water in order to verify our simulations against measurement data. Bremsstrahlung splitting of 20 and Russian roulette were used as global variance reduction techniques. The ECUT value was set to 0.521 MeV, and the PCUT was set to 0.01 MeV. For the cobalt machine, the measurement dataset used was the one from Ref. 14. For the LINAC, the measured data were acquired during commissioning. Agreement between measured and calculated data was obtained within less than 1% uncertainty.

Then, using the BEAM code, the phase‐space files of a $30\times 30\;{\text{cm}}^{2}$ were produced at the bottom surface of the secondary collimators for both machines. The phase‐space files for the irregular fields were created by simulating the blocks. (The same variance reduction and the same parameters were used as for the $10\times 10\;{\text{cm}}^{2}$ fields). The phase‐space files were used with DOSXYZ^(16)^ to calculate the dose distributions in two phantoms each of resolution 5 mm along the *x*‐ and *y*‐axes, and 10 mm along the *z*‐axis. The first phantom was made of water, and the second phantom had a slab of inhomogeneity of dimensions 10cm×10cm×5cm located 2 cm below the surface. The inhomogeneity represented either the lung equivalent or bone material. The results were analyzed at various depths, inside and below the inhomogeneity, for both simulations (lung and bone equivalent materials) and presented as correction factor graphs along the axis. The number of histories simulated gave a standard error of less than ±1% for all simulations.

The Batho method was used as the internal correction method in Eq. (8). Using the developed test code, we obtained the correction factors for the setup geometry of Fig. 4 at various depths along the *x*‐ and *y*‐axes for both beam energies. Our results were compared against those from the Monte Carlo calculations, with results obtained from the PLATO RTPS (Nucletron B.V., Veenendaal, the Netherlands) using ETAR and with results of the Batho not taking into account the field shape.

**Figure 5:**
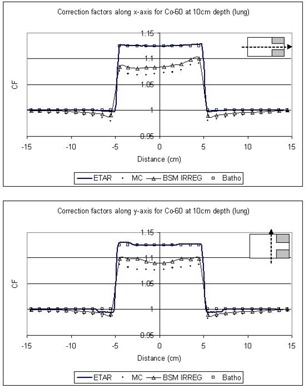
Comparison of the correction factors for lung inhomogeneity at a depth of 10 cm along the *x*‐ and *y*‐axes for the Co‐60 $30\times 30\;{\text{cm}}^{2}$ field

**Figure 6:**
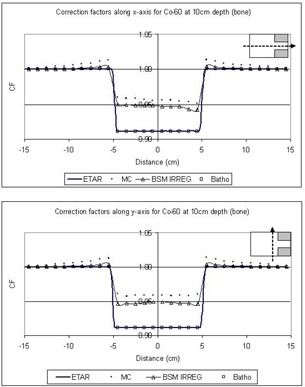
Comparison of the correction factors for bone inhomogeneity at a depth of 10 cm along the *x*‐ and *y*‐axes for the Co‐60 $30\times 30\;{\text{cm}}^{2}$ field

**Figure 7:**
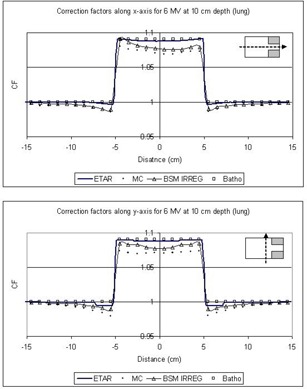
Comparison of the correction factors for lung inhomogeneity at a depth of 10 cm along the *x*‐ and *y*‐axes for the 6‐MV $30\times 30\;{\text{cm}}^{2}$ field

**Figure 8:**
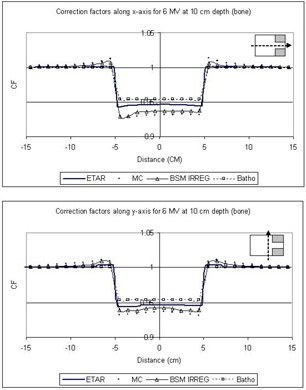
Comparison of the correction factors for bone inhomogeneity at a depth of 10 cm along the *x*‐ and *y*‐axes for the 6‐MV $30\times 30\;{\text{cm}}^{2}$ field

In Figs. 5 to 8 the correction factors are calculated along the *x*‐ and *y*‐axes for both photon beam energies at the depth of 10 cm for both lung and bone equivalent materials. The irradiating field is $30\times 30\;{\text{cm}}^{2}$ defined at SAD=100cm for both 6‐MV and Co‐60 photon beams. The SAD technique was used in our case; that is, the source‐to‐skin distance was set to 90 cm for all cases. The inhomogeneity was represented as a slab of dimensions $10\times 10\times 5\;{\text{cm}}^{3}$ placed symmetrically in the central beam axis and 2 cm under the surface. As mentioned before, the off‐axis effect is not taken into account because the beams are assumed to be flat and symmetrical over the entire irradiating field. The correction factors were obtained every 1 cm along both axes. The 3D BSM IRREG method correctly predicts the correction factor below the inhomogeneity and also at points where no inhomogeneity lies directly above them. When moving away from the interface and no inhomogeneity is above the calculation point, the correction factor is affected by the presence of the inhomogeneity. This is due to a lack or an excess of scatter from the inhomogeneity slab to points close to the interface, depending on whether ρ is lower or higher than $1\;g/{\text{cm}}^{3}$. Moving farther from the interface the phenomenon is less pronounced, and the correction factor approximates unity. Using the ETAR as the inhomogeneity correction algorithm in our commercial TPS, we found that there is no significant difference in our case when compared to the Batho method. This is in agreement with the results of du Plessis et al.,^(17)^ who found that the difference between Batho and ETAR is minimal for large fields. Also, we can see that Batho and ETAR do not predict the effect on the correction factor when the points are close to the “interface” of the inhomogeneity and the medium (Figs. 5 to 8). Furthermore, the results from ETAR appear to be the same as the those of the Batho method; the difference between the two methods is negligible. The difference between ETAR, Batho, and the 3D BSM IRREG is about 2.5% and is more obvious in the Co‐60 case (Figs. 5 and 6). Overall, we can see that the BSM IRREG results are in very good agreement with the Monte Carlo results; the discrepancy is less than 1.0% in most cases, which can be attributed to the limitations of the internal correction factor and to the Monte Carlo uncertainties.

We should also note that the addition of the two blocks in the field (as shown in Fig. 4) affects the correction factor by approximately 2%, depending on the location of the point of calculation and on the extent of the inhomogeneity. The original BSM method would fail to forecast this, since it is not able to account for the irregularity of the field. ETAR and Batho also fail to forecast this phenomenon, but 3D BSM IRREG predicts the correction factor more accurately than the other methods (*x*‐axis graphs in Figs. 5 to 8). This effect is more pronounced when considering the points in or under the inhomogeneity slab and toward the side of the field where the block is.

## IV. DISCUSSION

It is generally accepted that Monte Carlo algorithms can achieve the optimum dose calculation inside and/or in the proximity of heterogeneity, where there is analytical calculation of primary and scatter photons and electrons. Taking into account the complexity of calculations for clinical photon beams as well as the time limitation, there is only one TPS that directly uses Monte Carlo code for clinical cases, the PEREGRINE^(18)^ RTPS. Furthermore, according to our knowledge, the majority of the commercial RTPS use the ETAR method or another conventional heterogeneity correction algorithm. Thus, we believe that there is room for improvement in the existing conventional methods.

The original 3D‐BSM method was able to accurately predict the dose perturbation for rectangular fields when the inhomogeneity was not intersected by the beam axis. We propose a new method that is based on the 3D‐BSM and is able to predict the correction to the dose perturbation due to the presence of inhomogeneity for any given field shape. This is achieved by adapting the Clarkson method of sector integration to the 3D‐BSM algebraic summation of theoretical fields. The method cannot be applied under conditions of electronic disequilibrium, since it is dependent on the internal correction methods (such as the power law (Batho), etc.). It is in the same category as the original 3D‐BSM method of local deposition (no electron transport) with the ability to use 3D density sampling.

As we can see from Figs. 5 to 8, the ETAR and Batho methods break down at the interface where they could administer significant clinical underdosage in the case of lung and overdosage in the case of the bone. The proposed method provides an alternative to computing the dose more accurately with minimal input. It is also easily implemented.

Since 3D BSM IRREG is largely dependent on the internal correction method, $\left(C_{i}(\rho_{j},\;R_{j}\right)$, the current version using the Batho method of inhomogeneity correction can be applied to energies of photon beams up to 6 MV. This choice of energies was made because the majority of treatment techniques involving heterogeneities (e.g., lung) include low‐energy beams.^(19)^ Correction methods acceptable for higher energies (above 6 MV) such as the improved power law (Batho) method, which uses TPR instead of TAR or DTAR, etc., can be used and are expected to give good results.

The results of the method are found to be in good agreement with Monte Carlo simulations, and in most cases the discrepancy is less than 1.5%. Measurements were made using a solid water phantom and cork to mimic the geometry for a few selected points, in order to verify the Monte Carlo results. Again, the agreement was within the Monte Carlo uncertainty. The largest discrepancies are observed at the points that are close to the interfaces. This is mostly due to the lack of dosimetric data for very small fields (less than $4\times 4\;{\text{cm}}^{2}$) and very large fields (larger than $40\times 40\;{\text{cm}}^{2}$). Very small or very large fields are substituted by the smallest and largest available in the dataset, respectively.

The current implementation of the method can compute the correction factors for a plane of calculation at a time. For resolution of 1×1 mm (at the plane of the point of interest) and for a $20\times 20\;{\text{cm}}^{2}$ field it takes approximately 20 s on a 2‐GHz PC. Further development of the code should improve the calculation speed, introduce more “internal correction methods,” provide more dosimetric data, and allow the user to introduce his or her own dosimetric dataset. Also, improvement of the interpolation routines, for fields smaller and larger than those in the dosimetric dataset, will eliminate the source of error that appears mostly near the inhomogeneity interface. Furthermore, improvement in the dosimetric dataset in order to better treat the smaller field as proposed by Woo et al.^(11)^ would increase the accuracy of the algorithm, especially for the cases where small fields have to be calculated.

## V. CONCLUSION

The combination of the 3D‐BSM and Clarkson methods of sector integration allows us to propose a new method, which can generalize any conventional inhomogeneity correction method. In fact, the appropriate choice of a bulk method (in our case, the power law/Batho method) assures an acceptable correction for situations where the point of calculation lies off the beam axis, and the lateral extent of the inhomogeneity is smaller than the field size. This approach improves the basic inhomogeneity correction method by taking into account that (1) the point of calculation could be anywhere in the irradiating volume, (2) the primary arriving to the point of calculation may not be affected by the presence of the inhomogeneity, and (3) the shape of the field could be altered by the presence of shielding blocks. It is clear that the correction method proposed works also for cases where the point of interest lies under the inhomogeneity, and the lateral extent of the inhomogeneous medium is larger than the field size, that is, for all cases. In most of the cases investigated, the agreement between Monte Carlo results and the results from the computer application of the proposed method is less than 1.5%.

This new method can improve the accuracy in the prediction of the correction factor in the presence of irregular fields by a factor of 2% to 3% compared with the original BSM method. That is mostly because the original method fails to take into account the shape of the irradiating field. The 3D BSM IRREG method gives the same results as its ancestor and can be used instead.

## ACKNOWLEDGMENTS

We would like to thank the MDS‐Nordion for providing the data of the cobalt machine for our Monte Carlo calculations.
